# Bis(2,6-dimethyl­pyrazine-κ*N* ^4^)diiodidozinc(II)

**DOI:** 10.1107/S1600536808005382

**Published:** 2008-03-05

**Authors:** Sun Hwa Lee, Sea-Hyun Kim, Pan-Gi Kim, Cheal Kim, Youngmee Kim

**Affiliations:** a Department of Fine Chemistry and Eco-Products and Materials Education Center, Seoul National University of Technology, Seoul 139-743, Republic of Korea; b Tree Breeding Division, Korea Forest Research Institute, Suwon 441-350, Republic of Korea; c Department of Forest Resources and Environment, Kyungpook National University, Sangju 742-711, Republic of Korea; d Division of Nano Sciences, Ewha Womans University, Seoul 120-750, Republic of Korea

## Abstract

In the title compound, [ZnI_2_(C_6_H_8_N_2_)_2_], the Zn^II^ ion is coordinated by two iodide anions and two N atoms from 2,6-dimethyl­pyrazine in a distorted tetra­hedral geometry.

## Related literature

For background information, see: Batten & Robson (1998 ▶); Chi *et al.* (2006 ▶); Evans & Lin (2002 ▶); Hong *et al.* (2004 ▶); Janiak (2003 ▶); Janaik & Scharmann (2003 ▶); Kasai *et al.* (2000 ▶); Kitagawa *et al.* (2004 ▶); Luan *et al.* (2005 ▶, 2006 ▶); Moler *et al.* (2001 ▶); Moulton & Zaworotko (2001 ▶); Ryu *et al.* (2005 ▶); Wang *et al.* (2006 ▶); Blake *et al.* (1999 ▶); Saalfrank *et al.* (2001 ▶).
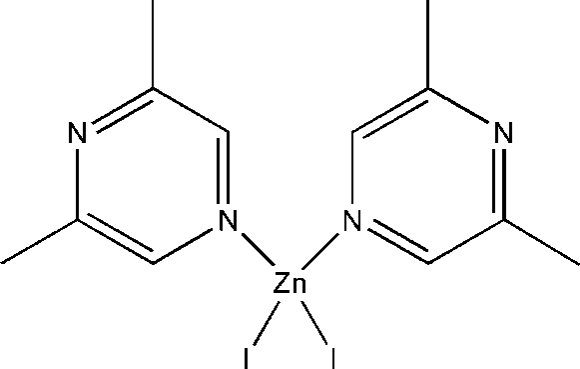


## Experimental

### 

#### Crystal data

[ZnI_2_(C_6_H_8_N_2_)_2_]
*M* *_r_* = 535.48
Monoclinic, 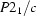

*a* = 9.1825 (7) Å
*b* = 13.8144 (10) Å
*c* = 13.6242 (10) Å
β = 98.381 (1)°
*V* = 1709.8 (2) Å^3^
*Z* = 4
Mo *K*α radiation
μ = 5.04 mm^−1^
*T* = 170 (2) K
0.10 × 0.05 × 0.05 mm

#### Data collection

Bruker SMART CCD diffractometer
Absorption correction: none
9413 measured reflections
3344 independent reflections
2518 reflections with *I* > 2σ(*I*)
*R*_int_ = 0.109

#### Refinement

*R*[*F* ^2^ > 2σ(*F* ^2^)] = 0.032
*wR*(*F* ^2^) = 0.069
*S* = 0.81
3344 reflections
176 parameters
H-atom parameters constrained
Δρ_max_ = 0.81 e Å^−3^
Δρ_min_ = −1.27 e Å^−3^

### 

Data collection: *SMART* (Bruker, 1997 ▶); cell refinement: *SAINT* (Bruker, 1997 ▶); data reduction: *SAINT*; program(s) used to solve structure: *SHELXS97* (Sheldrick, 2008 ▶); program(s) used to refine structure: *SHELXL97* (Sheldrick, 2008 ▶); molecular graphics: *SHELXTL* (Sheldrick, 2008 ▶); software used to prepare material for publication: *SHELXTL*.

## Supplementary Material

Crystal structure: contains datablocks I, global. DOI: 10.1107/S1600536808005382/dn2320sup1.cif

Structure factors: contains datablocks I. DOI: 10.1107/S1600536808005382/dn2320Isup2.hkl

Additional supplementary materials:  crystallographic information; 3D view; checkCIF report

## Figures and Tables

**Fig. 1.**
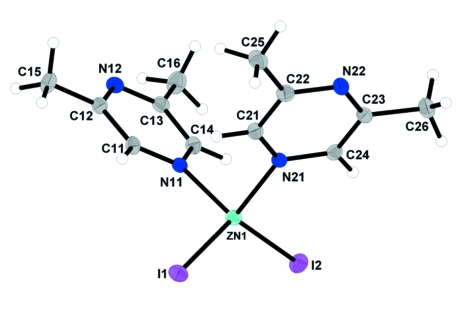
The structure of the title compound showing the atom-labeling scheme. Displacement ellipsoids are shown at the 30% probability level. H atoms are represented as small spheres of arbitrary radii.

## supplementary crystallographic information

### Comment

Much interest has recently been focused on the rational design and construction of novel discrete and polymeric metal-organic complexes, not only due to their structural and topological novelty (Batten & Robson, 1998, Moler *et al.*, 2001, Moulton & Zaworotko, 2001), but also for their potential applications as functional materials such as catalysis, molecular recognition, separation, and nonlinear optics (Hong *et al.*, 2004, Evans & Lin, 2002, Kasai *et al.* 2000, Kitagawa *et al.*, 2004). It has shown that many factors such as the coordination geometry of metal ions (Chi *et al.*,2006), the structure of organic ligands (Wang *et al.*,2006), the solvent system (Ryu *et al.*, 2005), the counteranion (Luan *et al.*, 2006), and the ratio of ligands to metal ions (Blake *et al.*, 1999, Saalfrank *et al.*, 2001) influence highly on the structure of metal-organic complexes. In addition, it has been considered that the secondary forces such as hydrogen-bonding, pi-pi stacking, and host–guest interactions are of importance as well (Luan *et al.*, 2005, Janaik & Scharmann, 2003, Janiak, 2003). For obtaining novel structural motifs with predictable properties, therefore, a large number of organic ligands were designed and utilized. Among them, 2,6-dimethylpyrazine was often selected. We have also reacted ZnI_2 _with 2,6-dimethylpyrazine to form a new zinc complex and report here on the crystal structure of diiodobis(2,6-dimethylpyrazine)zinc(II).

Asymmetric unit contains a whole molecule (Fig. 1). Zn^II^ ion is coordinated by two iodide anions and two nitrogen atoms from 2,6-dimethylpyrazine to form a distorted tetrahedral geometry (Fig. 1). Zn—I bond distances are 2.5393 (7) and 2.5442 (6) Å, and I—Zn—I and N—Zn—N bond angles are 122.78 (2) and 101.39 (14)°, respectively.

### Experimental

244.29 mg (0.75 mmol) of ZnI_2_ were dissolved in 4 ml water and carefully layered by 4 ml e thanol solution of 2,6-dimethylpyrazine ligand (165.52 mg, 1.5 mmol). Suitable crystals of the title compound for X-ray analysis were obtained in a few weeks.

### Refinement

(type here to add refinement details)

### Crystal data

**Table:**

[ZnI_2_(C_6_H_8_N_2_)_2_]	*F*(000) = 1008
*M**_r_* = 535.48	*D*_x_ = 2.080 Mg m^−^^3^
Monoclinic, *P*2_1_/*c*	Mo *K*α radiation, λ = 0.71073 Å
Hall symbol: -P 2ybc	Cell parameters from 2888 reflections
*a* = 9.1825 (7) Å	θ = 2.7–25.6°
*b* = 13.8144 (10) Å	µ = 5.04 mm^−^^1^
*c* = 13.6242 (10) Å	*T* = 170 K
β = 98.381 (1)°	Rod, colorless
*V* = 1709.8 (2) Å^3^	0.10 × 0.05 × 0.05 mm
*Z* = 4	

### Data collection

**Table:**

Bruker SMART CCD diffractometer	2518 reflections with *I* > 2σ(*I*)
Radiation source: fine-focus sealed tube	*R*_int_ = 0.109
graphite	θ_max_ = 26.0°, θ_min_ = 2.1°
φ and ω scans	*h* = −11→11
9413 measured reflections	*k* = −17→16
3344 independent reflections	*l* = −9→16

### Refinement

**Table:**

Refinement on *F*^2^	Primary atom site location: structure-invariant direct methods
Least-squares matrix: full	Secondary atom site location: difference Fourier map
*R*[*F*^2^ > 2σ(*F*^2^)] = 0.032	Hydrogen site location: inferred from neighbouring sites
*wR*(*F*^2^) = 0.069	H-atom parameters constrained
*S* = 0.81	*w* = 1/[σ^2^(*F*_o_^2^) + (0.0147*P*)^2^] where *P* = (*F*_o_^2^ + 2*F*_c_^2^)/3
3344 reflections	(Δ/σ)_max_ = 0.001
176 parameters	Δρ_max_ = 0.81 e Å^−^^3^
0 restraints	Δρ_min_ = −1.28 e Å^−^^3^

### Special details

**Table:**

Geometry. All e.s.d.'s (except the e.s.d. in the dihedral angle between two l.s. planes) are estimated using the full covariance matrix. The cell e.s.d.'s are taken into account individually in the estimation of e.s.d.'s in distances, angles and torsion angles; correlations between e.s.d.'s in cell parameters are only used when they are defined by crystal symmetry. An approximate (isotropic) treatment of cell e.s.d.'s is used for estimating e.s.d.'s involving l.s. planes.
Refinement. Refinement of *F*^2^ against ALL reflections. The weighted *R*-factor *wR* and goodness of fit *S* are based on *F*^2^, conventional *R*-factors *R* are based on *F*, with *F* set to zero for negative *F*^2^. The threshold expression of *F*^2^ > σ(*F*^2^) is used only for calculating *R*-factors(gt) *etc*. and is not relevant to the choice of reflections for refinement. *R*-factors based on *F*^2^ are statistically about twice as large as those based on *F*, and *R*- factors based on ALL data will be even larger.

### Fractional atomic coordinates and isotropic or equivalent isotropic displacement parameters (Å^2^)

**Table:**

	*x*	*y*	*z*	*U*_iso_*/*U*_eq_	
Zn1	0.07870 (6)	0.24556 (4)	0.76694 (4)	0.02508 (15)	
I1	0.07868 (4)	0.15009 (3)	0.60725 (2)	0.03357 (11)	
I2	0.19801 (4)	0.41101 (3)	0.79495 (2)	0.03516 (11)	
N11	−0.1362 (4)	0.2579 (3)	0.7987 (3)	0.0244 (9)	
N12	−0.4102 (4)	0.2602 (3)	0.8610 (3)	0.0290 (10)	
N21	0.1716 (4)	0.1540 (3)	0.8789 (3)	0.0247 (9)	
N22	0.3046 (4)	0.0275 (3)	1.0237 (3)	0.0283 (10)	
C11	−0.2359 (5)	0.1893 (4)	0.7695 (3)	0.0262 (11)	
H11	−0.2115	0.1387	0.7276	0.031*	
C12	−0.3750 (5)	0.1903 (4)	0.7992 (3)	0.0274 (11)	
C13	−0.3120 (5)	0.3282 (4)	0.8896 (3)	0.0280 (12)	
C14	−0.1739 (5)	0.3274 (4)	0.8575 (3)	0.0278 (11)	
H14	−0.1058	0.3778	0.8781	0.033*	
C15	−0.4843 (5)	0.1130 (4)	0.7682 (4)	0.0361 (13)	
H15A	−0.5043	0.0768	0.8267	0.054*	
H15B	−0.4450	0.0688	0.7222	0.054*	
H15C	−0.5758	0.1422	0.7351	0.054*	
C16	−0.3539 (5)	0.4070 (4)	0.9555 (4)	0.0438 (15)	
H16A	−0.4566	0.4258	0.9340	0.066*	
H16B	−0.2898	0.4631	0.9515	0.066*	
H16C	−0.3429	0.3837	1.0241	0.066*	
C21	0.1310 (5)	0.0616 (4)	0.8802 (3)	0.0278 (12)	
H21	0.0566	0.0385	0.8299	0.033*	
C22	0.1950 (5)	−0.0025 (4)	0.9535 (3)	0.0302 (12)	
C23	0.3442 (5)	0.1193 (4)	1.0219 (3)	0.0277 (12)	
C24	0.2793 (5)	0.1839 (4)	0.9502 (3)	0.0265 (11)	
H24	0.3112	0.2494	0.9515	0.032*	
C25	0.1460 (6)	−0.1051 (4)	0.9560 (4)	0.0391 (14)	
H25A	0.1946	−0.1436	0.9098	0.059*	
H25B	0.0390	−0.1085	0.9366	0.059*	
H25C	0.1720	−0.1308	1.0234	0.059*	
C26	0.4667 (6)	0.1544 (4)	1.1002 (3)	0.0406 (14)	
H26A	0.4261	0.1714	1.1606	0.061*	
H26B	0.5133	0.2115	1.0756	0.061*	
H26C	0.5401	0.1030	1.1152	0.061*	

### Atomic displacement parameters (Å^2^)

**Table:**

	*U*^11^	*U*^22^	*U*^33^	*U*^12^	*U*^13^	*U*^23^
Zn1	0.0246 (3)	0.0250 (3)	0.0258 (3)	0.0006 (2)	0.0044 (2)	0.0019 (3)
I1	0.0398 (2)	0.0359 (2)	0.02578 (18)	0.00179 (16)	0.00728 (14)	−0.00169 (16)
I2	0.0342 (2)	0.0259 (2)	0.0448 (2)	−0.00311 (15)	0.00379 (16)	0.00327 (16)
N11	0.023 (2)	0.027 (2)	0.023 (2)	0.0031 (18)	0.0014 (17)	0.0060 (19)
N12	0.028 (2)	0.034 (3)	0.026 (2)	0.005 (2)	0.0062 (18)	0.001 (2)
N21	0.025 (2)	0.027 (3)	0.0217 (19)	−0.0010 (18)	0.0039 (16)	0.0008 (19)
N22	0.028 (2)	0.030 (3)	0.026 (2)	0.002 (2)	0.0014 (18)	0.000 (2)
C11	0.022 (3)	0.028 (3)	0.027 (2)	0.004 (2)	−0.001 (2)	−0.002 (2)
C12	0.023 (3)	0.029 (3)	0.028 (3)	0.003 (2)	0.000 (2)	0.007 (2)
C13	0.029 (3)	0.030 (3)	0.025 (3)	0.004 (2)	0.004 (2)	0.003 (2)
C14	0.028 (3)	0.026 (3)	0.028 (3)	0.001 (2)	0.000 (2)	0.000 (2)
C15	0.024 (3)	0.038 (3)	0.045 (3)	−0.006 (3)	0.003 (2)	−0.008 (3)
C16	0.032 (3)	0.049 (4)	0.051 (3)	0.001 (3)	0.009 (3)	−0.011 (3)
C21	0.029 (3)	0.028 (3)	0.026 (3)	−0.003 (2)	0.004 (2)	−0.004 (2)
C22	0.032 (3)	0.029 (3)	0.030 (3)	0.000 (2)	0.008 (2)	−0.001 (2)
C23	0.030 (3)	0.032 (3)	0.021 (2)	−0.003 (2)	0.004 (2)	−0.004 (2)
C24	0.023 (2)	0.029 (3)	0.029 (3)	−0.001 (2)	0.008 (2)	−0.002 (2)
C25	0.050 (3)	0.030 (3)	0.034 (3)	−0.002 (3)	−0.003 (3)	0.007 (3)
C26	0.041 (3)	0.045 (4)	0.033 (3)	−0.008 (3)	−0.006 (2)	0.002 (3)

### Geometric parameters (Å, °)

**Table:**

Zn1—N21	2.068 (4)	C15—H15A	0.9800
Zn1—N11	2.088 (4)	C15—H15B	0.9800
Zn1—I2	2.5393 (7)	C15—H15C	0.9800
Zn1—I1	2.5442 (6)	C16—H16A	0.9800
N11—C14	1.328 (6)	C16—H16B	0.9800
N11—C11	1.337 (6)	C16—H16C	0.9800
N12—C13	1.321 (6)	C21—C22	1.398 (7)
N12—C12	1.351 (6)	C21—H21	0.9500
N21—C21	1.331 (6)	C22—C25	1.489 (7)
N21—C24	1.346 (5)	C23—C24	1.392 (6)
N22—C23	1.321 (6)	C23—C26	1.513 (6)
N22—C22	1.348 (6)	C24—H24	0.9500
C11—C12	1.395 (7)	C25—H25A	0.9800
C11—H11	0.9500	C25—H25B	0.9800
C12—C15	1.485 (7)	C25—H25C	0.9800
C13—C14	1.401 (7)	C26—H26A	0.9800
C13—C16	1.496 (7)	C26—H26B	0.9800
C14—H14	0.9500	C26—H26C	0.9800
			
N21—Zn1—N11	101.39 (14)	H15B—C15—H15C	109.5
N21—Zn1—I2	108.49 (11)	C13—C16—H16A	109.5
N11—Zn1—I2	107.19 (12)	C13—C16—H16B	109.5
N21—Zn1—I1	105.22 (11)	H16A—C16—H16B	109.5
N11—Zn1—I1	109.72 (10)	C13—C16—H16C	109.5
I2—Zn1—I1	122.78 (2)	H16A—C16—H16C	109.5
C14—N11—C11	117.7 (4)	H16B—C16—H16C	109.5
C14—N11—Zn1	121.4 (3)	N21—C21—C22	121.8 (4)
C11—N11—Zn1	120.5 (3)	N21—C21—H21	119.1
C13—N12—C12	118.6 (4)	C22—C21—H21	119.1
C21—N21—C24	117.6 (4)	N22—C22—C21	120.3 (5)
C21—N21—Zn1	120.7 (3)	N22—C22—C25	118.2 (4)
C24—N21—Zn1	121.7 (3)	C21—C22—C25	121.5 (5)
C23—N22—C22	117.5 (4)	N22—C23—C24	122.5 (4)
N11—C11—C12	121.5 (5)	N22—C23—C26	118.2 (4)
N11—C11—H11	119.2	C24—C23—C26	119.2 (5)
C12—C11—H11	119.2	N21—C24—C23	120.3 (5)
N12—C12—C11	120.0 (5)	N21—C24—H24	119.9
N12—C12—C15	118.6 (4)	C23—C24—H24	119.9
C11—C12—C15	121.3 (5)	C22—C25—H25A	109.5
N12—C13—C14	120.8 (5)	C22—C25—H25B	109.5
N12—C13—C16	118.1 (4)	H25A—C25—H25B	109.5
C14—C13—C16	121.1 (5)	C22—C25—H25C	109.5
N11—C14—C13	121.4 (5)	H25A—C25—H25C	109.5
N11—C14—H14	119.3	H25B—C25—H25C	109.5
C13—C14—H14	119.3	C23—C26—H26A	109.5
C12—C15—H15A	109.5	C23—C26—H26B	109.5
C12—C15—H15B	109.5	H26A—C26—H26B	109.5
H15A—C15—H15B	109.5	C23—C26—H26C	109.5
C12—C15—H15C	109.5	H26A—C26—H26C	109.5
H15A—C15—H15C	109.5	H26B—C26—H26C	109.5
